# A comparative magnetic resonance spectroscopy study of GABA+ and glutamate referenced to creatine and phosphocreatine in the left dorsolateral prefrontal cortex of perimenopausal women and women of reproductive age

**DOI:** 10.3389/fpsyt.2022.989050

**Published:** 2022-10-26

**Authors:** Kim H. Tran, Jessica Luki, Sarah Hanstock, Christopher C. Hanstock, Peter Seres, Katherine Aitchison, Tami Shandro, Jean-Michel Le Melledo

**Affiliations:** ^1^Department of Psychiatry, University of Alberta, Edmonton, AB, Canada; ^2^Department of Biomedical Engineering, University of Alberta, Edmonton, AB, Canada; ^3^Department of Medical Genetics, University of Alberta, Edmonton, AB, Canada; ^4^Neuroscience and Mental Health Institute, University of Alberta, Edmonton, AB, Canada; ^5^Women and Children’s Research Institute, University of Alberta, Edmonton, AB, Canada; ^6^Division of Clinical Sciences, Psychiatry Section, Northern Ontario School of Medicine, Thunder Bay, ON, Canada; ^7^Lois Hole Hospital for Women, Royal Alexandra Hospital, Edmonton, AB, Canada

**Keywords:** perimenopause, magnetic resonance spectroscopy (MRS), glutamate, GABA, left dorsolateral prefrontal cortex (DLPFC), depression

## Abstract

**Objective:**

The perimenopause is associated with an increased risk of developing a major depressive (MD) episode. The biological changes occurring during perimenopause responsible for this increased risk of depression remain to be elucidated. Postmortem and magnetic resonance spectroscopy (MRS) studies have revealed decreased gamma-aminobutyric acid (GABA) and glutamate (Glu) levels in the dorsolateral prefrontal cortex (DLPFC) of MD patients. The objective of this study was to compare LDLPFC GABA+ and Glu ratios (referenced to creatine and phosphocreatine) in healthy reproductive-aged (RD) and perimenopausal (PM) women.

**Materials and methods:**

Eighteen healthy PM and 20 RD women were included in the study. Our dependent variables, LDLPFC Glu and GABA+ ratios which include homocarnosine and macromolecules, were measured *via* MRS, using a 3 Tesla magnet. Absence of current or past psychiatric diagnosis was confirmed *via* a structured interview. RD participants were scanned during the early follicular phase of the menstrual cycle (MC). PM women were scanned outside of ovulatory cycles.

**Results:**

Mean LDLPFC GABA+ and Glu ratios were not statistically different between the PM group and RD group (PM mean = 0.10 ± 0.06, RD mean = 0.11 ± 0.04, *t* = –0.383, *df* = 36, *d* = −0.13, *p* = 0.70) (PM mean = 0.56 ± 0.06, RD mean = 0.57 ± 0.05, *t* = –0.794, *df* = 36, *d* = −0.26, *p* = 0.43), respectively. The perimenopause demarcates the end of the reproductive life. Unsurprisingly PM women were older than RD women (PM women: 48.8 ± 3.55 years, range 41–53 years old; RD women: 31.5 ± 9.66 years, range 18–47 years old) (*p* < 0.001). This inherent entanglement of group and age is a limitation of our study.

**Conclusion:**

Contrary to our previous findings of decreased GABA+ and Glu in the medial prefrontal cortex in perimenopausal women, the perimenopause is not associated with decreased GABA+ or Glu ratios in the LDLPFC. This suggests that brain areas playing a role in MD display different sensitivity to the female hormones fluctuations associated with perimenopause.

## Introduction

The perimenopause is associated with an increased risk of developing a major depressive (MD) episode. The prevalence rate of MD has been described to be between two-to-fourteen times higher during the perimenopause than during reproductive years (1). There is a dearth of knowledge regarding the impact of perimenopause on the brain. Investigating the change in brain biology associated with perimenopause will contribute to the elucidation of the cerebral mechanisms responsible for the increased risk of MD during the perimenopause. Hormonal fluctuations associated with perimenopause are thought to be responsible for this increased risk, albeit the exact physiological mechanism responsible for this remains unclear (2). Key neurotransmitters in specific brain areas involved in the pathophysiology of MD such as gamma-aminobutyric acid (GABA) and glutamate (Glu) in the medial prefrontal cortex (MPFC) and the dorsolateral prefrontal cortex (DLPFC) are primary candidates for studying the increased risk of MD during the perimenopause. We have already shown that GABA+ and Glu referenced to creatine (Cr) and phosphocreatine (PCr) are decreased in the MPFC of perimenopausal women (3, 4). We propose now to investigate GABA and Glu in the other major brain area involved in depressive symptomatology, i.e., the DLPFC.

Perimenopause (PM) is a phase within the normal female reproductive life cycle characterized by the cessation of the menstrual cycle (MC) regularity. The average age of onset is 46 years old, with a duration of approximately 5 years before the transition into menopause (5). During perimenopause, before the total cessation of production by the ovaries, estrogen concentrations decrease erratically with successive increases and decreases, while its counterpart, progesterone, declines in a more gradual manner (6). The importance of the medial prefrontal cortex (MPFC) and the dorsolateral prefrontal cortex (DLPFC) in mood regulation/emotional processing and the pathophysiology of major depression (MD) have been well-established (7).

Several studies have highlighted the importance of GABA and Glu in the pathophysiology of MD (8–11). GABA, the main inhibitory neurotransmitter of the central nervous system, is widespread throughout the brain, and it is estimated that 60–75% of all synapses are GABAergic (10). Glu on the other hand, is the major excitatory neurotransmitter, and its excitatory activity counterbalances GABA’s inhibitory activity. Gamma-aminobutyric acid (GABA) and glutamate (Glu) are among the most promising avenues of biological research in major depression and the faster acting antidepressants have been linked to these two neurotransmitters (12–14).

Magnetic resonance spectroscopy (MRS) is the sole non-invasive neuroimaging technique that enables *in vivo* detection and measurement of brain metabolites such as GABA and Glu in localized brain regions (15). Decreased GABA+ and Glu referenced to creatine + phosphocreatine (Cr+PCr) in the MPFC and the DLPFC of MD patients have been observed in MRS investigations (10). However, there have been multiple sources of variability in the MRS investigations of the GABAergic and glutamatergic system in MD. Many MRS studies of Glu in fact reported on Glx, the combined signal of Glu and glutamine (Gln).

7T MRS investigations provide a clear spectral resolution of Glu and Gln, allowing for the individual measurement of Glu (16). However, our group developed an optimized PRESS using in-house simulations, which allowed for the measurement of Glu with minimal contamination from other metabolite peaks based on the work of Harris et al. (17, 18). Similarly, GABA alone has been difficult to measure individually and most MRS research investigation in MD have reported on GABA+ (a combination of GABA, homocarnosine and macromolecules) (19). Due to similar chemical shift (resonant frequencies of an atomic nucleus referenced to a standard compound) GABA is contaminated with signals from homocarnosine (Hcar) and macromolecules (MM), therefore, GABA will be referred to as GABA+ in this paper. In our investigation, MEscher-GArwood Point RESolved Spectroscopy (MEGA-PRESS) is the spectral difference method used to isolate GABA+ signal.

Another source of heterogeneity of the MRS investigations of MD has been the molecule of reference used. Most of these studies have, however, used Cr+PCr as a molecule of reference. The location and the size of the voxels can also contribute to sources of variability. However, several prefrontal cortex voxels have been the main targets of investigation of Glu (13, 20, 21), with the occipital cortex being the voxel of interest for the studies of GABA due to technological convenience (12, 22).

Besides magnet strength, MRS technique, the choice of the normalization reference and the brain region of interest, other factors can impact the results of a MRS investigation in MD. For instance, little attention has been paid to the reproductive status of these women and the phase of the menstrual cycle or the perimenopausal status. This is an issue since perimenopausal status (3, 4) and phase of menstrual cycle have been shown to affect brain Glu and GABA (17, 23). For example, we have shown that MPFC Glu/Cr+PCr are decreased in the luteal phase (LP) compared to the follicular phase of healthy controls (18). Epperson et al. observed that healthy menstruating women experienced a significant decrease in occipital GABA from the follicular phase to the late LP (23). We have also shown that GABA+/Cr+PCr and Glu/Cr+PCr are decreased in the MPFC of perimenopausal women (3, 4).

It is important to note that the left DLFPC (LDLPFC) is the voxel of interest in this paper instead of the right DLPFC because previous PET studies that assessed cerebral glucose metabolic rates, have shown that the LDLPFC is more hypo-activated in MD patients (7, 24). Several studies have also highlighted the efficacy of left DLPFC transcranial magnetic stimulation in treating refractory MD (25).

The large objective of this MRS study was to assess whether decreased LDLPFC GABA+ and Glu ratios occurring during the perimenopause could be possible contributors to the increased risk of development of depression during the perimenopause. Our hypothesis was that LDLPFC GABA+ and Glu ratios, our dependent variables, would be decreased in perimenopausal women compared to healthy women of reproductive age (RD). Since GABA and Glu are found mostly in the grey matter (GM) and that GM decreases with age, we hypothesized that the decrease in GABA+ and Glu ratios in the inherently older PM women will persist independent of brain tissue composition. We will also test whether there are correlations between female hormone measurements and LDLPFC GABA+ and Glu ratios. Our investigation is the first MRS investigation of GABA+ and Glu in the LDLPFC of perimenopausal woman.

## Materials and methods

### Participants

Eighteen perimenopausal and 20 reproductive-aged physically and mentally healthy women were recruited for the study. All participants were 18 years of age and older. The study protocol was approved by the Health Research Ethics Board of the University of Alberta and conducted in accordance with the Declaration of Helsinki. A pre-screening telephone interview was first conducted. Written informed consent was collected from all eligible participants. Following collection of informed consent, participants took part in two sessions: a screening interview and a scanning visit.

#### Inclusion criteria, both age groups

Physically and mentally healthy women, who were 18 years of age and older, and used a birth control method that did not deliver female hormones.

#### Inclusion criteria, reproductive-aged group

Regular occurrence of menstrual cycle (MC).

#### Inclusion criteria, perimenopause group

Undergoing perimenopause, with menopausal status being defined as either early perimenopausal (menstrual bleeding had occurred in the past 3 months with changes in frequency over the last 12 months), or late perimenopausal (no menstrual bleeding within the past 3 months but some menstruation within the last 12 months). This classification is recommended by the World Health Organization and the Stages of Reproductive Aging Workshop (26, 27).

#### Exclusion criteria for all subjects

(1) Current or lifetime history of any psychiatric illness, confirmed using the Mini-International Neuropsychiatric Interview (MINI Version 7.0.2) based on Diagnostic and Statistical Manual of Mental Disorders-5 criteria (28, 29); (2) any contraindications to MRI; (3) pregnancy; (4) use of birth control methods which deliver female hormone; (5) any medical condition that would interfere with the study, e.g., endocrine or neurological condition (30); and (6) intake of medications that may impact brain GABA or Glu function at any time while participating in the study (31). Of note no participants were taking any medications for the duration of the study.

### Study protocol

After completing the phone interview, participants who appeared to be eligible for the study were scheduled for a screening session. During this interview, a complete medical and psychiatric history were completed. The MINI was used to screen for psychiatric illnesses (28, 29). Participants who met the inclusion and exclusion criteria were then booked for a scanning visit. The scanning visit was completed between day two and six of the follicular phase (FP) of the MC for RD women and early PM women (but during an anovulatory MC). Late PM women were scanned after 3 months of anovulatory cycles. Early and late PM women were contacted later to ensure that they did not have any menses during the following month.

All participants underwent an MRS scan and completed the Beck Depression Inventory (BDI). PM women were additionally administered the Greene Climacteric Scale (GCS) and Menopause Rating Scale (MeRS) to evaluate their PM-related symptomology. A blood sample measuring plasma estradiol and progesterone was collected from all participants. Third generation Elecsys^®^ immunoassay (Roche Diagnostics) was used to measure plasma estradiol, while Access Progesterone assay (Beckman Coulter) was used to measure plasma progesterone.

### Magnetic resonance spectroscopy and imaging

Magnetic resonance data were collected at the Peter S. Allen MR Research Centre, University of Alberta, Edmonton, Canada, using a Siemens Prisma 3 Tesla (T) scanner (Erlangen, Germany) equipped with a 64-channel head-neck coil for signal reception. Anatomical images were acquired using sagittal 3D T1-weighted magnetization-prepared rapid gradient-echo (MPRAGE) sequence in 3 min 39 s (TR: 1800 ms, TE: 2.37 ms, TI: 900 ms, flip angle: 8, field of view: 250 × 250 mm, image matrix: 288 × 288, slice thickness: 0.85 mm, number of slices: 208, resolution: 0.87 × 0.87 × 0.85 mm, parallel acceleration factor: 3). These images were used for planning the position of spectroscopy voxels as well as for volumetric and segmentation analysis.

**FIGURE 1:**
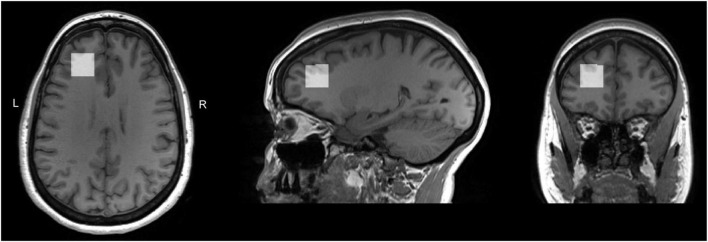
T1 weighted images for a sample subject with overlaid LDLPFC MRS voxel. Voxel position and orientation were prescribed so that the voxel was perpendicular to the midline in transverse and coronal views and parallel to the corpus callosum line in a sagittal view; the voxel was then placed, using all three planes of the anatomical image, above the horn of the lateral ventricles, anterior, as far lateral as possible while remaining in the cortex. Care was taken to avoid any lipid/skull contamination.

Voxel position and orientation were positioned so that the voxel was perpendicular to the midline in transverse and coronal views and parallel to the corpus callosum line in a sagittal view; the voxel was then placed, using all three planes of the anatomical image, above the horn of the lateral ventricles, anterior, as far lateral as possible while remaining in the cortex. Care was taken to avoid any lipid/skull contamination (Figure 1). Identical voxel size (20 × 20 × 20 mm^3^) and position was used for both PRESS and MEGA-PRESS acquisitions.

**FIGURE 2:**
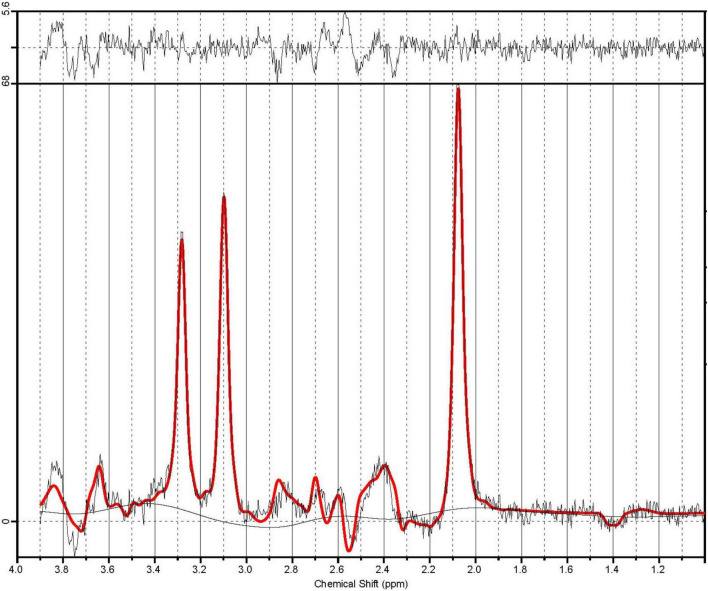
A sample PRESS spectrum with sequence timings optimized for recovering signal from glutamate (PRESS TE1,2 = 90.80 ms). The spectra in illustrate the unfiltered data superimposed with the LCModel fit in red. The residual noise is shown above.

A customized version of the PRESS sequence, allowing for asymmetric echo times was used to measure spectrum for Glu and Cr+PCr. Data were acquired summing 64 averages, repeated twice, in 5 min 45 s (TR: 2500 ms, TE1,2: 90, 18 ms, BW: 2000 Hz, delta frequency: −2.4 ppm, 2048 spectral data points). A sample PRESS spectrum is shown in Figure 2. Automated metabolite quantification of the proton MR spectra was performed using LCModel^®^ (version 6.3-1L) (17).

**FIGURE 3:**
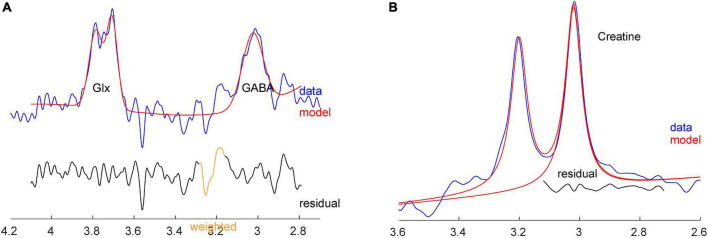
**(A)** Sample GABA+ and Glx spectra with fit from Gannet, from the same subject. **(B)** Sample Cr spectrum with fit from Gannet.

MEGA-PRESS, implemented as Siemens “work in progress” WIP859F, was used to measure the spectrum for GABA and Cr+PCr analysis (17, 32). A sample MEGA-PRESS spectrum is shown in Figures 3A,B. Adequate signal-to-noise ratio was obtained by summing 320 averages composed of 160 pairs where the editing pulses were either on or off, in 10 min 56 s (MEGA-PRESS: TR: 2000 ms, TE: 68 ms, BW: 2000 Hz, editing pulse frequency: 1.9 ppm, delta frequency: 1.7 ppm, 2,048 spectral data points). Automated metabolite quantification of the proton MR spectra was performed using Gannet software (version 3.0^1^) on saved raw data (.DAT files), providing relative concentrations of GABA+ and Cr+PCr. No unsuppressed water spectra were acquired.

Manual and quantitative inspection was conducted on each spectrum fitted by both LCModel and Gannet to assure quality with respect to line-shape, line-width (full width at half maximum (FWHM)), signal-to-noise ratio (SNR) and quality of fit. Spectra not meeting these quality check criteria or those with an LCModel fit with a standard deviation > 15% were excluded from further analysis. Gannet data were accepted if SNR > 80 and FWHM < 10 Hz. MRS quality is particularly sensitive to participant motion; the Gannet pipeline automatically excludes samples for which the frequency offset deviates sufficiently to affect the spectrum quality (i.e., it excludes unusable metabolite data because of brief movements during the scanning period). The number of averages that were automatically excluded were similar between the two groups (number of excluded averages PM mean = 0.75% ± 0.48%, RD mean = 1.30% ± 1.49%, with maximum excluded averages for one participant = 17, out of 320 averages acquired).

Magnetic resonance spectroscopy data for one RD and two PM participants were severely affected by motion; the RD and early PM participant were scanned again between day two and six of FP, whereas the late PM woman was scanned outside of anovulatory cycle (confirmed by a phone call 1 month post-scan) consistent with study protocol.

Statistical Parametric Mapping (SPM12) (33) were used for T1 image volumetric and segmentation analysis. Included in Gannet package pipeline were steps to generate a mask of the MRS voxel in T1-image space and to utilize the SPM12 “Segmentation” function to calculate relative GM, WM, and CSF fractions within MPFC voxel (later referenced to as %GM, %WM, and %CSF).

### Statistical analysis

For all statistical tests, the level of significance was defined to be *p* ≤ 0.05. Statistical analysis was performed using the IBM Statistical Package for Social Sciences software for Windows Version 26.0 (SPSS 26.0) (IBM Corp., Armonk, NY, USA). LDLPFC GABA+ and Glu ratios were our dependent variables. The data in this study were normally distributed and the results reported reflect combining the total sample size, i.e., both PM and RD women together, unless stated otherwise. A two-tailed *t*-test was used for independent sample analysis of variables between PM and RD women. Additionally, covariate analysis was performed where %GM content was treated as a covariate. Metabolite data was analyzed using Cr+PCr as a reference molecule (Figure 3).

In order to analyze the impact age had on LDLPFC GABA+ and Glu ratios, simple linear regression was utilized. Multiple regression analysis controlling for age and %GM was conducted to test whether these factors significantly affected LDLPFC GABA+ and Glu ratios Cross tabulation analysis of Eta coefficient was used to determine strength of association between Participant group and Age. One-way analysis of variance (ANOVA) test was used to compare the mean of our dependent variables, LDLPFC GABA+ and Glu ratios between groups. A One-way analysis of covariance (ANCOVA) was conducted to determine a statistically significant difference between PM and RD women on LDLPFC GABA+ and Glu ratios, controlling for age, LDLPFC %GM and %WM.

The Pearson correlation coefficient (PCC) was used to analyze the relationship between LDLPFC GABA+/Glu ratios and age, LDLPFC GABA+ and Glu ratios and female hormone concentrations, and BDI scores and group. PCC was also used to analyze the relationship between BDI, GCS, and MeRS scores and mean LDLPFC GABA+ and Glu ratios in the PM group.

## Results

### Two tailed *t*-test

**FIGURE 4:**
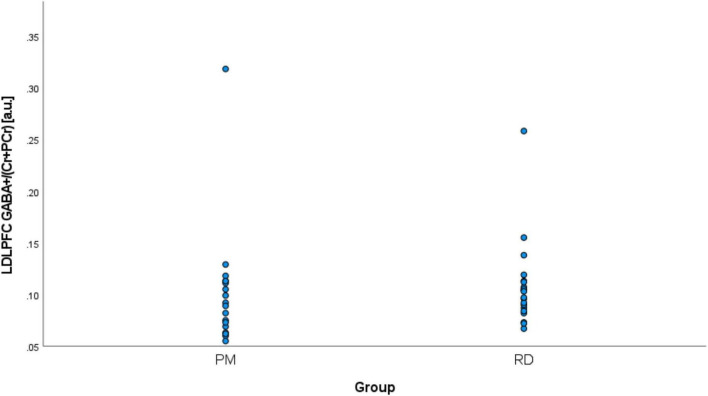
Comparison of LDLPFC creatine and phosphocreatine-referenced GABA+ levels in healthy perimenopausal and reproductive-aged women. GABA+, gamma-aminobutyric acid including homocarnosine and macromolecules; LDLPFC, left dorsolateral prefrontal cortex; PM, healthy perimenopause; RD, healthy reproductive-aged.

**FIGURE 5:**
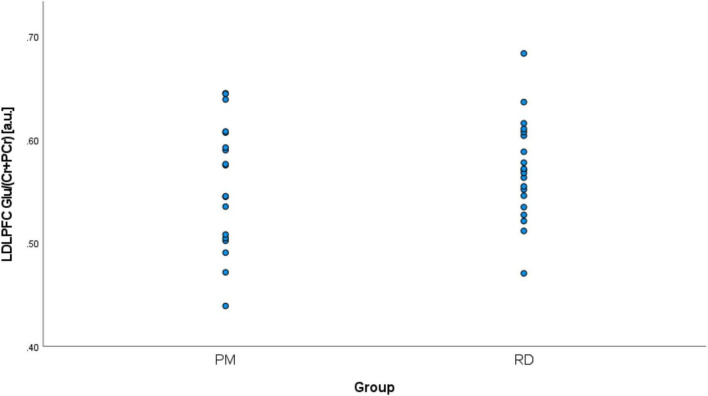
Comparison of LDLPFC creatine and phosphocreatine-referenced Glu levels in healthy perimenopausal and reproductive-aged women. Glu, glutamate; LDLPFC, left dorsolateral prefrontal cortex; PM, healthy perimenopause; RD, healthy reproductive-aged.

Perimenopausal women (48.8 ± 3.55 years, range 41–53 years old) were significantly older than RD participants (31.5 ± 9.66 years, range 18–47 years old) (*p* < 0.001). LDLPFC GABA+ and Glu ratios (relative to Cr+PCr) were not statistically different between PM and RD participants [LDLPFC GABA+: 0.10 ± 0.06, 0.11 ± 0.04, *p* = 0.70, *d* = −0.13, 95% CI (−0.79, 0.54); LDLPFC Glu: 0.56 ± 0.06, 0.57 ± 0.05, *p* = 0.43, *d* = −0.26, 95% CI (−0.92, 0.40)], respectively (Table 1 and Figures 4, 5).

**TABLE 1:** Creatine and phosphocreatine-referenced GABA+ and Glu concentrations and brain tissue composition within the LDLPFC of healthy PM and RD women.

	PM participants (*n* = 18)		RD participants (*n* = 20)		Group	
			
	Mean	SD	Mean	SD	*p*-value	t(d.f. = 36)
**Metabolite**						
GABA+	0.101	0.06	0.108	0.04	0.704	−0.383
Glu	0.556	0.06	0.570	0.05	0.432	−0.794
%GM	28.74	4.84	29.36	4.34	0.679	−0.418
%WM	67.80	6.02	68.86	4.88	0.552	−0.600
%CSF	3.46	2.03	1.78	0.92	0.004*	3.237

GABA+, GABA including homocarnosine and macromolecules; Glu, glutamate; GM, gray matter; MPFC, medial prefrontal cortex; PM, perimenopausal; RD, reproductive; WM, white matter. Brain metabolite measured in institutional units. Metabolite concentration referenced to creatine and phosphocreatine. *A significant difference between groups.

Since brain tissue composition of the voxel may affect GABA+ and Glu ratios, we compared brain tissue composition between the two groups. %GM and %WM were not significantly different between PM and RD women [%GM: 28.74 ± 4.84, 29.36 ± 4.33, *p* = 0.68, *d* = −0.14, 95% CI (−0.80, 0.53); %WM: 67.80 ± 6.02; 68.86 ± 4.88, *p* = 0.55, *d* = −0.20, 95% CI (−0.86, 0.47)] (Table 1). %CSF was greater in PM women compared to RD women [%CSF: 3.46 ± 2.03; 1.78 ± 0.92, *p* = 0.004, *d* = 0.96, 95% CI (0.38, 1.54)], respectively (Table 1).

Baseline estradiol [PM: 237.89 ± 280.33; RD: 146.15 ± 53.98; *p* = 0.19, *d* = 0.46, 95% CI (−0.25, 1.17)] and progesterone [PM: 1.74 ± 0.84; RD: 1.79 ± 1.15; *p* = 0.89, *d* = −0.05, 95% CI (−0.71, 0.62)] concentrations were not significantly different between groups.

Although PM patients had higher mean BDI scores (3.67 ± 2.89; range: 10; low score: 0; high score: 10) than RD women (1.45 ± 1.79; range: 6; low score: 0; high score: 6) [*p* = 0.009, *d* = 0.85, 95% CI (0.23, 1.48)], scores for both groups were within the range corresponding to scores that are considered normal mood fluctuations in life. This suggests that participants were not experiencing any clinically significant depressive symptomology. In addition, mean GCS (5.17 ± 4.55) and MeRS (5.22 ± 4.31) scores indicated that PM participants were experiencing little or no PM-related symptomology (RD women were not administered these questionnaires).

### Simple linear regression

Simple linear regression was performed to analyze the impact age had on LDLPFC GABA+ and Glu ratios. Age explained approximately 4% of the observed variance in LDLPFC GABA+ and Glu ratios observed [LDLPFC GABA+: *R*^2^ = 0.038, *F*_(1,36)_ = 1.44, *p* = 0.24; LDLPFC Glu: *R*^2^ = 0.042, *F*_(1,36)_ = 1.57, *p* = 0.22]. This analysis also showed that age did not significantly predict concentrations of GABA+ or Glu ratios (LDLPFC GABA+: β_1_ = −0.001, *p* = 0.24; LDLPFC Glu: β_1_ = −0.001, *p* = 0.22).

### Multiple regression

A multiple regression was run to predict LDLPFC GABA+ and Glu ratios from age and LDLPFC %GM. These variables did not significantly predict LDLPFC GABA+/Glu ratios (relative to Cr+PCr): *F*_(2,35)_ = 1.065, *p* = 0.355, *R*^2^ = 0.057; *F*_(2,35)_ = 1.476, *p* = 0.242, *R*^2^ = 0.078, respectively.

### Cross tabulation analysis

Cross tabulation analysis of Eta coefficient was used to determine strength of association between Participant group and Age. Eta coefficient was found to be 0.77, which suggest a strong association between Participant group and Age.

### One-way analysis of variance

Knowing that Participant group and Age were strongly associated, ANOVA analysis without age correction was used to determine if LDLPFC GABA+ and Glu ratios were significantly different between PM and RD women. We found no significant differences between groups [LDLPFC GABA+: *F*_(1,36)_ = 0.147, *p* = 0.70; LDLPFC Glu: *F*_(1,36)_ = 0.631, *p* = 0.43].

### A one-way analysis of covariance

Based on the ANCOVA analysis, there was no significant effect of reproductive status on LDLPFC GABA+/Glu ratios, after controlling for age, LDLPFC %GM and %WM: *F*_(1,35)_ = 0.092, *p* = 0.74; *F*_(1,35)_ = 0.511, *p* = 0.48, respectively (Tables 2–5). Small effect sizes were observed for both LDLPFC GABA+ and Glu, η^2^ = 0.003 and 0.014, respectively.

**TABLE 2:** Unadjusted and covariate adjusted descriptive statistics for LDLPFC GABA+/Cr+PCr.

Group	Unadjusted			Adjusted	
		
	N	Mean	SEMean	Mean	SEMean
Perimenopause	18	0.101	0.014	0.102	0.012
Reproductive	20	0.108	0.011	0.107	0.011

**TABLE 3:** Analysis of covariance for LDLPFC GABA+/Cr+PCr by group with brain tissue composition as a function of age (Age, LDLPFC %GM and LDLPFC %WM) as covariates.

Source	SS	df	MS	*F*	*p*	η^2^
Brain tissue composition (covariate)	0.005	1	0.005	1.954	0.171	0.053
Group	0.000	1	0.000	0.092	0.764	0.003
Error	0.086	35	0.002			

*R* Squared = 0.057 (Adjusted *R* Squared = 0.003).

**TABLE 4:** Unadjusted and covariate adjusted descriptive statistics for LDLPFC Glu/Cr+PCr.

Group	Unadjusted			Adjusted	
		
	N	Mean	SEMean	Mean	SEMean
Perimenopause	18	0.556	0.015	0.557	0.013
Reproductive	20	0.570	0.011	0.570	0.012

**TABLE 5:** Analysis of covariance for LDLPFC Glu/Cr+PCr by group with brain tissue composition as a function of age (Age, LDLPFC %GM and LDLPFC %WM) as covariates.

Source	SS	df	MS	*F*	*p*	η ^2^
Brain tissue composition (covariate)	0.007	1	0.007	2.369	0.133	0.063
Group	0.001	1	0.001	0.511	0.480	0.014
Error	0.017	35	0.003			

*R* Squared = 0.080 (Adjusted *R* Squared = 0.027).

### Pearson correlation coefficient

Correlational analysis showed that LDLPFC GABA+ and Glu ratios were not significantly related to age, with a weak negative relationship (LDLPFC GABA+: *r* = −0.20, *p* = 0.24; LDLPFC Glu: *r* = −0.20, *p* = 0.22).

**FIGURE 6:**
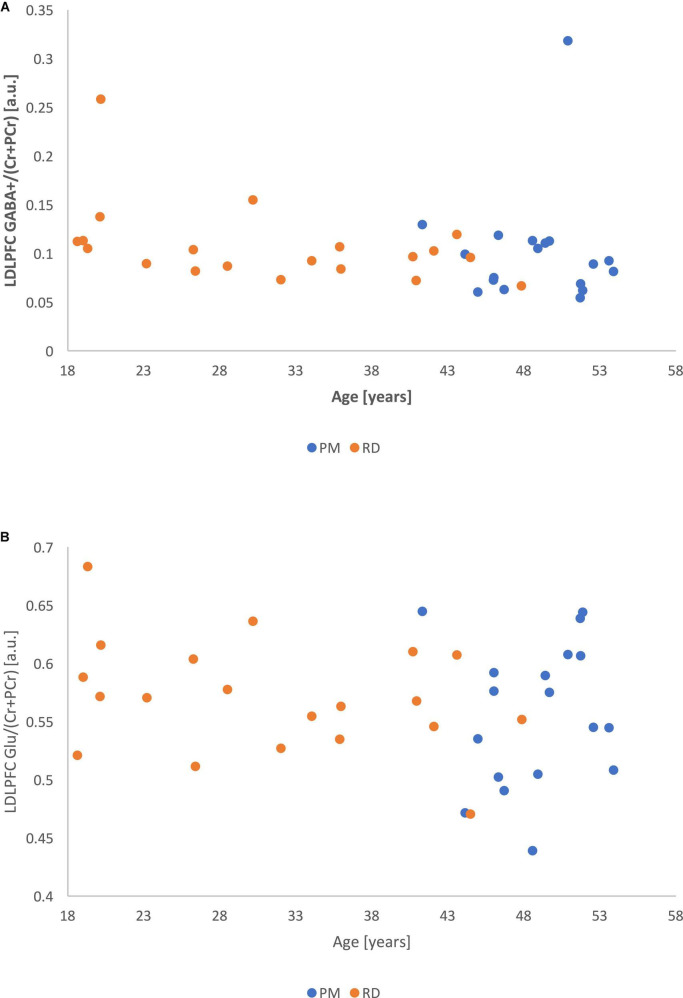
**(A)** Metabolite ratios of LDLPFC GABA+ (referenced to Cr+PCr) as a function of age. **(B)** Metabolite ratios of LDLPFC Glu (referenced to Cr+PCr) as a function of age.

There was no significant relationship between age and %GM (*r* = −0.17, *p* = 0.30) (Figures 6A,B).

Correlational analysis revealed that LDLPFC GABA+ and Glu ratios were not significantly correlated to either baseline estradiol (LDLPFC GABA+: *r* = −0.26, *df* = 36, *p* = 0.12; LDLPFC Glu: *r* = −0.21, *p* = 0.20) or progesterone (LDLPFC GABA+: *r* = 0.00, *p* = 0.99; LDLPFC Glu: *r* = −0.08, *p* = 0.62) concentrations.

Further correlational analyses were conducted between BDI, GCS, and MeRS scores and LDLPFC GABA+ and Glu ratios in the PM group. These analyses revealed no significant correlation between BDI and LDLPFC GABA+ ratios: *r* = 0.13, *p* = 0.62; and LDLPFC Glu ratios: *r* = −0.34, *p* = 0.17; GCS and LDLPFC GABA+ ratios: *r* = −0.10, *p* = 0.71 and LDLPFC Glu ratios: *r* = −0.16, *p* = 0.54; or MeRS scores and LDLPFC GABA+ ratios: *r* = −0.19, *p* = 0.45 and LDLPFC Glu ratios: *r* = −0.18, *p* = 0.48. LDLPFC GABA+ and Glu ratios, respectively, in the PM group.

### Outliers analysis

We noticed two outliers in regard to LDLPFC GABA+/Cr+PCr. One PM and one RD participant had much higher GABA+/Cr+PCr than the other participant in their respective group (Figure 4). After exclusion of these two outliers, the difference of GABA+ ratios remained non-significant: [LDLPFC GABA+: 0.09 ± 0.02, 0.10 ± 0.02, *p* = 0.15, *d* = −0.48, 95% CI (−1.15, 0.19)].

In regard to the analysis of estradiol and progesterone measurements, we have performed two statistical analyses based on the fact that some hormonal measurements were non-detectable, i.e., <30 pmol/L for estradiol and <1.0 nmol/L for progesterone. In the first analysis, we have replaced the measurements that were not detectable by the threshold limit of detection, 30 pmol/L for estradiol and 1.0 nmol/L for progesterone. One PM woman had non-detectable levels of estradiol whereas no RD woman had undetectable levels of estradiol. As for progesterone, 4 PM women had undetectable levels and 8 RD women had undetectable levels. In the second statistical analysis, we have excluded the women with non-detectable hormonal measurements and found: estradiol [PM: 250.12 ± 283.97; RD: 146.15 ± 53.98; *p* = 0.16, *d* = 0.52, 95% CI (−0.22, 1.26)] and progesterone [PM: 1.95 ± 0.84; RD: 2.31 ± 1.34; *p* = 0.39, *d* = −0.34, 95% CI (−1.16, 0.47)] concentrations remain not significantly different between groups. Correlational analysis revealed that LDLPFC GABA+ and Glu ratios were not significantly correlated to either baseline estradiol (LDLPFC GABA+: *r* = −0.27, *p* = 0.11; LDLPFC Glu: *r* = −0.22, *p* = 0.18) or progesterone (LDLPFC GABA+: *r* = −0.08, *p* = 0.72; LDLPFC Glu: *r* = −0.15, *p* = 0.47) concentrations, even when excluding these participants.

## Discussion

This is the first study comparing LDLPFC GABA+ and Glu in healthy PM women and RD women. We showed that LDLPFC GABA+ and Glu ratios did not differ between healthy PM and RD women. This suggests that contrary to MPFC GABA+ and Glu ratios (3, 4), LDLPFC GABA+ and Glu ratios are unaffected by the female hormonal fluctuations associated with perimenopause. These findings do not support our underlying suggestion that changes in LDLPFC GABA+ and Glu are a candidate dysregulation for the increased risk of depression during the perimenopause.

Since PM women are inherently older than RD women, the impact of age is therefore difficult to disentangle from perimenopausal status. GABA+ and Glu are mainly found in GM (34) and full-brain fractional GM declines with age (35). %GM between PM and RD women were similar, which suggest the lack of a substantial difference between both groups. In addition, multiple regression analysis controlling for age and %GM revealed that these two factors did not significantly contribute to the concentrations of LDLPFC GABA+ or Glu ratios. Furthermore, when we controlled age, LDLPFC %GM, and %WM as covariates (which we combined and denoted as the variable Brain Tissue Composition in Tables 2–5), our ANCOVA results suggested that reproductive status still had no significant effect on LDLPFC GABA+ and Glu ratios. A small effect size was also observed for these ratios.

We found significant differences in %CSF between PM and RD women, which is corroborated by previous neuroimaging normative studies on the effects of aging on the human brain (36).

This study combined with our prior research suggest that MPFC GABA+ and Glu ratios but not LDLPFC GABA+ and Glu ratios are affected by female hormonal fluctuations. The higher connection of the MPFC to the subcortical regions that express a high concentration of estrogen receptor alpha (ERα), estrogen receptor beta (ERβ) and G protein-coupled estrogen receptor (GPER), i.e., amygdala, hippocampus, and thalamus, may explain the discrepancies between the two brain areas (37–39).

As expected we found a lack of substantial difference in estrogen and progesterone concentrations between PM and RD women. Indeed, these hormone levels (due to their variability), are of little help in determining perimenopause status clinically. Accordingly, we saw a greater standard deviation in estradiol levels in PM women compared to RD women. We were also unable to find a significant correlation between concentrations of estradiol and progesterone and LDLPFC GABA+ and Glu ratios in the current study. Female hormones and their metabolites, also have a delayed impact on transcription (40), which would not be captured by female hormone measurements concomitant to the scanning.

It is possible that the design of our study impacted our results. We scanned participants during early FP to minimize differences in hormone concentrations, as such are relatively low and stable at this time compared to other phases of the MC. Different results might have been obtained if scanning had occurred during a different phase of the MC. For instance, Epperson et al. have shown fluctuations of GABA ratios during the MC in healthy reproductive controls (23).

A limitation of this study was that sample sizes were relatively small for both PM (*n* = 18) and RD (*n* = 20) groups. However, we were able to show decreased GABA+ and/or Glu ratios in similar or smaller samples that investigated the impact of reproductive status on these metabolites (3, 18, 33). We do not believe that our negative results are explained by a small sample size and therefore by a type II error. The total sample size needed to show that the difference in Glu/Cr+PCr and GABA/Cr+PCr we observed in the LDLPFC is statistically significant at a power of 0.8 is 786 and 786, respectively, which suggests that this difference is not meaningful.

Of the 18 PM participants in total, 12 were in early PM while six were in late PM. There is a greater risk of experiencing MD in late PM compared to early PM (41). However, due to the small sample size of PM women, we were unable to perform meaningful comparisons of LDLPFC GABA+ and Glu ratios between early PM and late PM women. Future studies with greater numbers of women in the late PM should be conducted.

There are also limitations related to the MRS techniques we used. While MEGA-PRESS is a robust spectral difference method, it is difficult to purely obtain GABA resonance without contamination from MM and Hcar, since the latter metabolites share similar chemical shifts as GABA (19). While our 3T technique allows for a satisfactory discrimination of Glu and Glx concentrations, a 7T magnet would allow a better separation of the peaks of Glu and Glx.

Another limitation of the study is the inherent entanglement of group and age with RD women being younger than PM women. Further investigations including inherently older menopausal women as controls could help assessing the impact of age in the MPFC and LDLPFC measurement of GABA+ and Glu ratios during the perimenopause.

Reported LDLPFC GABA+ and Glu ratios would be affected by changes in the concentration of Cr+PCr. However, Cr+PCr have been used extensively as a reference molecule in previous MRS MD research (42). In addition, our group recently demonstrated that Cr+PCr was unaffected by the large female hormone changes experienced during pregnancy and postpartum (33, 43). Together, these findings indicate that Cr+PCr is a viable reference molecule for MRS research in PM women.

We assume that the reported LDLPFC GABA+ and Glu ratios are related to GABAergic and glutamatergic neurotransmission. However, Carbon-13 (^13^C) MRS is the only *in vivo* noninvasive method that allows for differentiation of GABAergic/glutamatergic neurotransmission and cell-specific energetics with signaling and non-signaling purposes (44). As a result, it is difficult to determine the source of our GABA+ and Glu measurements in the LDLPFC because it can either be neurotransmission related or neuronal metabolism related, or both. For the same reasons, ^13^C MRS concomitant measurements of both GABA and Glu would also be necessary to assess the balance of GABA and Glu neurotransmission in PM women.

## Conclusion

In summary, the major finding of our study is that in contrast to our findings of decreased MPFC Glu and GABA+ ratios in the perimenopause, LDLPFC GABA+ and Glu ratios are not significantly different between healthy PM women and healthy RD women. This suggests that the GABA and Glu measurements in brain areas playing a role in MD are differently affected by the fluctuations of female hormones occurring during the perimenopause.

## Data availability statement

The raw data supporting the conclusions of this article will be made available by the authors, without undue reservation.

## Ethics statement

The studies involving human participants were reviewed and approved by University of Alberta Health Research Ethics Board. The patients/participants provided their written informed consent to participate in this study.

## Author contributions

KT contributed to investigation, data analysis, methodology, project administration, writing, and revision of the original draft. JL contributed to investigation, data collection, project administration, methodology, reviewing, and editing the manuscript. SH contributed to investigation, reviewing, and editing the manuscript. CH contributed to funding acquisition, data interpretation, reviewing, and editing the manuscript. PS contributed to investigation, data analysis, reviewing, and editing the manuscript. TS contributed to funding acquisition, investigation, reviewing, and editing the manuscript. KA contributed to investigation, data interpretation, graduate student funding, reviewing, and editing the manuscript. J-ML contributed to writing, editing the manuscript, funding acquisition, conceptualization, and supervision. All authors contributed to the article and approved the submitted version.

## Footnotes

1
http://www.gabamrs.com
